# COVID-19 in Relation to Chronic Antihistamine Prescription

**DOI:** 10.3390/microorganisms12122589

**Published:** 2024-12-13

**Authors:** Anna Puigdellívol-Sánchez, Marta Juanes-González, Ana Calderón-Valdiviezo, Helena Losa-Puig, Roger Valls-Foix, Marta González-Salvador, Celia Lozano-Paz, Josep Vidal-Alaball

**Affiliations:** 1 Medicina de Familia, CAP Anton de Borja-Centre Universitari, c/Marconi-Cantonada Edison s/n, Consorci Sanitari de Terrassa (CST), 08191 Rubí, Spain; mjuanes@cst.cat (M.J.-G.); acalceronv@cst.cat (A.C.-V.); helenalosa@uic.es (H.L.-P.); rvalls@cst.cat (R.V.-F.); clozano@cst.cat (C.L.-P.); 2 Human Anatomy and Embryology Unit, Faculty of Medicine, c/Casanova 143, Universitat de Barcelona, 08036 Barcelona, Spain; 3 Hospital Álvaro Cunqueiro, Estrada de Clara Campoamor 341, 36213 Vigo, Spain; 4 Management, Control and Information Analysis Unit, Hospital de Terrassa, Consorci Sanitari de Terrassa (CST), Carretera de Torrebonica s/n, 08227 Terrassa, Spain; mgonzalezs@cst.cat; 5 Intelligence for Primary Care Research Group, Foundation University Institute for Primary Health Care Research Jordi Gol i Gurina, 08242 Manresa, Spain; jvidal.cc.ics@gencat.cat; 6 Unitat de Recerca i Innovació, Gerència d‘Atenció Primària i a la Comunitat de la Catalunya Central, Institut Català de la Salut, 08242 Manresa, Spain

**Keywords:** COVID-19, antihistamines, hospital admission, infection rate, death rate, polypharmacy

## Abstract

No hospitalizations or deaths occurred in residents with the COVID-19 infection, treated with antihistamines and azithromycin, of two external nursing homes during the first wave. We assessed whether patients receiving chronic antihistamines in our institution showed better clinical evolution. COVID-19 admissions and related deaths in the public Hospital of Terrassa (*n* = 1461) during the pandemic period (11 March 2020–5 May 2023) and cases (*n* = 32,888) during the period of full suspicion diagnosis (1 June 2020–23 March 2022) were referred to as the number of chronic treatments (nT) including or not including antihistamines (AntiHm or NOAntiHm), and their vaccination status before the first infection (VAC or NoVAC) in our assigned population (*n* = 140,681 at March 2020) was recorded. No deaths occurred in patients treated with up to ≤6 nT in the AntiHm group in all ages. A significant reduction in hospital admission was observed in the 2–7 nT groups either below or over 60 years old [Odds Ratio (OR) NoAntiHm/AntiHm = 1.76–1.32, respectively, in NoVAC or VAC (OR = 2.10 overall] and in the older ≥8 nT group (OR = 2.08 in NoVac]. In conclusion, patients with chronic antihistamine prescriptions, alone or with polypharmacy, showed reduced hospital admission and mortality rates, suggesting the safety of antihistamine treatment and the need to confirm its effectiveness in a prospective trial.

## 1. Introduction

Mortality rates in Spain saw a sudden and dramatic threefold increase in March 2020. According to the Health Ministry’s mortality monitoring service [1], this surge occurred two weeks after the COVID-19 outbreak in Italy [2] and four months after the initial outbreak in China, positioning Spain as the third country to experience a large-scale outbreak during the first wave of the pandemic.

Nursing homes were affected dramatically, reaching mortality rates of up to 48% among those who tested positive [3,4,5]. A study of the experience in a nursing home in Yepes (Toledo, Spain) described that all 84 residents (48% over 80 years old) tested positive after the first wave, but that no hospital admissions or deaths occurred after they had received treatment with antihistamines and azithromycin [6]. After applying the same treatment to another 468 COVID-19 patients infected between March 2020 and August 2021 in that area, the hospitalization rate fell significantly (approximately halved) compared to the official overall rates for Spain [7].

Other potential interventions to treat the disease were proposed early in the pandemic, including the use of angiotensin-converting enzyme inhibitors or the influenza vaccine. The influenza virus and coronavirus use similar strategies through hemagglutinin esterases to engage sialoglycans at the surface of target cells [8,9]. Primary evidence confirmed those hypotheses [8,9,10,11,12,13,14,15] but it was not supported by our preliminary findings [16], although we related higher COVID-19 mortality rates to polypharmacy and comorbidity [16,17].

Our previous results showed that 96% of mortality in hospital-admitted patients during the first wave was concentrated in patients over 60 years old, with a great mortality (>50%) in the most comorbid group (with a comorbidity Charlson index of 3.0 ± 2.7) taking more than eight chronic treatments, compared to 23% of them having just one chronic treatment (Charlson index = 0.5 ± 0.7), and 0% in the group without chronic conditions, that received no treatments (Charlson index of 0). Among patients suffering just one pathology, 30% presented hypertension, about 20% presented obesity, thyroid, or mental disorders, while less than 10% suffered neurological disorders, osteoporosis, gastric and colon pathologies or chronic obstructive pulmonary diseases. Comorbidity was associated with polypharmacy, and not with a specific pharmacologic group, since up to 91 different pharmacologic drugs were taken by the patients who were admitted early until the peak of the first wave [16].

We have recently published how the number of chronic treatments is related to hospitalization during all the COVID-19 waves and COVID-19 variants [17]. Frail patients are affected markedly by the COVID-19 infection [18] and polypharmacy is considered a risk predictor of COVID-19 severity [19] among nursing home residents [20].

This descriptive study was designed to confirm if patients suffering from a COVID-19 infection receiving chronic antihistamine treatment showed a better clinical evolution to support a future prospective study that might confirm the protective role of antihistamines.

## 2. Materials and Methods

The original descriptive study of patients admitted to a hospital with COVID-19 was approved by the Ethics Committee of Consorci Sanitari de Terrassa (CST) on 8 April 2020, (ref 02-20-161-021) and the observational clinical trial was posted on 29 April (NCT 04367883). The inclusion of antihistamines and amantadine in relation to the population of reference was approved on 13 June 2022 (ref 02-22-151-060) and posted on 17 August 2022 (https://clinicaltrials.gov/study/NCT05504057 (accessed on 1 December 2024)). The planning, conducting and reporting of the study were in line with the principles of the Declaration of Helsinki and the UE Regulation 2016/679.

### 2.1. Cases and Hospital Admissions

Anonymized data of COVID-19 hospital admissions during the pandemic (*n* = 1461; 11 March 2020 to 5 May 2023) and cases with confirmed diagnoses (*n* = 32,888, from 1 June 2020 to 3 March 2022) within the assigned CST population (*n* = 140,660 in March 2020) were analyzed. Infection, hospital admission, and mortality rates were related to the number of chronic treatments (nT) at admission, including treatment with or without antihistamines (AntiHm or NoAntiHm), COVID-19 vaccination status (having received at least one dose) before the first infection (VAC or NoVAC pre-infection), gender, and age. Deceased COVID-19 hospital-admitted patients were revised for (nT) and their COVID vaccination status before the first infection, under the terms of the UE Regulation 2016/679.9i. COVID-19 infection was confirmed either by polymerase chain reaction, antigen testing or clinical criteria (i.e., compatible interstitial pneumonia).

Suspected non-hospitalized cases from before 1 June 2020, reported cases after 23 March 2022 and COVID-19 hospital admissions from populations outside the CST were excluded from the comparison.

### 2.2. Socioeconomic Environment

The CST is an integral institution, a free public health consortium located in the North Metropolitan Barcelona Health Region, that covers seven primary healthcare centers, one long-term care center, and the Hospital of Terrassa.

The socioeconomic characteristics of the population of reference were obtained from the public open data of the Generalitat de Catalunya [21], which governs the healthcare in this area. The institution where the study was performed, the CST, covers a population with very different socioeconomic situations. Open data about the impact of the socioeconomic situation of the population in all of the 398 ‘ABS’ in Catalonia (‘Àrea Bàsica de Salut’—Basic Health Area-) were published in 2015 and included the percentages of people with annual incomes below 18,000 € or over 100,000 €, together with its impact on their life expectancy [22]. ABS Rubí 2, Rubí 3, Terrassa A, B, F belong to the CST. The rural village of Castellbisbal (11,767 habitants in 2024) and the residential village of Matadapera (9899 habitants in 2023) were assigned by the Health Department to the ABS Rubí 2 and Terrassa F, respectively, for management reasons. Rubí and Terrassa are industrial and commercial cities of 78,549 and 222,576 habitants, respectively, located in the metropolitan area of Barcelona.

The data of the healthcare area assigned to the CST, related to the three ABS with higher and lower life expectancy in Catalonia, are detailed in Table 1.

### 2.3. Statistics

OpenEpi (Open-source Epidemiologic Statistics for Public Health) [23] was used for data analysis. Descriptive tables with the assigned population’s vaccination status, gender, age, antihistamine treatment and stratified by the number of chronic treatments were prepared (Table 2 and Table 3).

Chi-square tests were used to compare the rate of infection (Table 4), hospitalization and death (Table 5 and Table S1), depending on the vaccination status prior to the first infection, stratified by the number of chronic treatments (including or not including antihistamines). Significant results are indicated in the corresponding table by a *.

A Benjamini–Hochberg correction for multiple comparisons has been also calculated and indicated in the comparison tables by a ‘+’ symbol beside * [24,25].

## 3. Results

The baseline characteristics related to vaccination prior to the first infection, polypharmacy, gender, and average age per subgroup are presented in Table 2. No significant differences were found between subgroups.

### 3.1. Vaccination and Infections

The full data of vaccination during the pandemic period, both prior or after the first infection, related to gender, nT and chronic antihistamine prescription, are presented in Table 3.

The VAC rate increased with the nT, being 37.2% in patients with 0 nT, 52.4% for those with 1 nT, 73.9% for those with 2–7 nT, and 91.6% in those with ≥8 nT (*p* < 0.001). There were no differences in the proportions of males and females with VAC, except in the 0 nT group (32.2% of VAC in males vs. 40.1% in females, *p* < 0.001). The VAC rate in patients over 60 years old exceeded 91.7% for overall patients with nT ≥ 2 but decreased for those younger than 60 years old with nT 2–7 (57.6–56–57.6%) or ≥8 (71.4–78.6%) for both the AntiHm and NoAntiHm groups, respectively (*p* < 0.001).

The overall rates of NoVAC/VAC for infection, hospitalization, and death were 1.69, 1.83, and 1.91, being of 1.7–3.4 and 4.2, respectively, for those taking at least one chronic treatment (*p* < 0.001).

The odds ratio (OR) of cases during the period of full diagnoses, between patients receiving or not receiving chronic treatment with antihistamines, stratified by the number of chronic treatments and vaccination status prior to the first infection, are presented in Table 4.

In the NoVAC group, the OR NoAntiHm/AntiHm infection rate for 1 nT was 1.14 (*p* = 0.06) and 0.70 in the VAC group (0.0001). The infection rate increased with nT in the NoVAC group (*p* = 0.001): 28% of infections with 0 nT, increasing progressively to 46% (8 nT). The infection rate was inverse in the VAC group (20% for 8 nT compared to 11% of 0 nT overall), both in the NOAntiHm group (19% for 8 nT vs. 10%) and in the AntiHm group (27% in 1 nT vs. 12% in 8 nT) (*p* < 0.001).

### 3.2. Hospital Admission and Death

The rates of hospital admission and COVID death in COVID-hospitalized patients during the pandemic period in patients treated or not treated with antihistamines, together with the OR between the two groups, depending on the vaccination status prior to the first infection, are presented in Table 5 and Supplementary Materials Table S1 (showing comparisons for patients over and below 60 years old).

The NOAntiHm/AntiHm admission ratio was significant in the 2–7 nT group [2.54 (NoVAC, *p* = 0.0008)–2.10 (VAC, *p* = 0.03)] and also in nT ≥ 8 [1.61 (NoVac, *p* = 0.03)–1.40 (VAC, *p* ≥ 0.11)].

No deaths occurred in patients treated with up to ≤6 nT in the AntiHm group. The VAC-NOAntiHm/AntiHm death ratio was 1.41 (2–7 nF)–1.19 (≥8 nF) (*p* > 0.05) and was significant in the NoVAC group [4.12 in 2–7 nT (*p* = 0.03) and 2.11 in nT ≥ 8 (*p* = 0.01)].

## 4. Discussion

This is the first study, to our knowledge, describing the association between chronic antihistamine prescription and the reduction in hospital admissions and in a large and well-defined population, corresponding to an integral public healthcare population that include primary care centers and their referral hospital. We are also unaware of any other studies describing the progressive infection rate related to polypharmacy in non-vaccinated patients.

### 4.1. Clinical Evidence

The approximate halving of the hospital admission and mortality rates is consistent with the experience in primary care [7] and in a nursing home in Yepes (Toledo, EU) [6]. However, the patients from that study with mild symptoms received azytromicin while severe patients also received levofloxacin 500/12 h, bronchodilators like mepifilin and prednisone in the case of breathing difficulty, which have also demonstrated its role in early treatment [26]. Azithromizin has been shown to increase interferons in cells from chronic obstructive pulmonary disease in vitro [27] and also, to target newly budded progeny viruses from the host cells while inactivating their endocytic activity; its intranasal administration successfully reduced the A(H1N1) pdm09 viral load in the lungs [28]. The current study did not include azithromycin treatment for patients admitted to the Hospital of Terrassa, precluding the assessment of the role of combined prescriptions.

Among COVID-19 patients, in the studies on antihistamines in primary care in the same zone of Yepes, showing reduced hospitalization and death, 46% received cetirizine because of its safety profile and low rates of side effects and interactions, but 35.7% received dexchlorpheniramine and 1.7% received ebastine [7]. In the CST, about 100 primary care physicians and allergologists were prescribing antihistamines, without any specific preference. This suggests that the better clinical evolution quantified may be related to a histamine class-effect due to several underlying mechanisms, including neuroprotective ligand receptors [29]; this also explains the lack of post-COVID-19 syndrome complaints in primary care patients treated with antihistamines [7].

The Pubmed evidence of the antihistamine effect from randomized controlled trials is scarce and limited to four articles on 15 November 2024 [30,31,32,33]. Tranilast, a mast cell inhibitor, increased O_2_ saturation, reduced long hospitalization and ICU deaths [30], while nasal azelastine (an antiH1 receptor antagonist) was related to early negativization [31]. Those assessing famotidine’s effects as an H2 receptor antagonist showed an improvement in the rate of symptom resolution [32], a higher score in the MoCA scale when assessing cognitive function and a larger reduction in the Hamilton Depression Rating Scale [33]. Some authors have described an increased short- and long-term response to IFN alfa stimulation, suggesting that famotidine can increase the anti-viral state of non-infected cells, thereby potentially increasing viral resistance [34]. However, other studies did not find consistent significant effects for famotidine, but reduced rates of diagnosis, hospitalization and death were detected for other common maintenance drugs like ACEI or ARB [35], in consonancy with our preliminary findings [16]. Further evidence in prospective controlled trials is needed.

### 4.2. Other Evidences

The protective effect of antihistamines suggested by our results is also consistent with other epidemiological reports [36] and in vitro studies [36,37]. It has been hypothesized that the activation of histamine pathways would lead to the cytokine storm in COVID-19 pathogenesis. Thus, antihistamine drugs could modulate the immune response [38].

It is interesting to note that in the early stages of the pandemic, 26 SARS-CoV-2 proteins were cloned, and human proteins physically associated with each were identified, with 332 high-confidence SARS-CoV-2–human protein–protein interactions. Among those, 66 druggable human proteins or host factors that could be targeted by 29 FDA-approved drugs were identified [39]. FDA-approved drugs that could be considered for the treatment of SARS-CoV-2 included the antihistamine drug Loratadine, a Histamine 1 receptor antagonist and Entacapone, a drug approved for Parkinson’s disease, acting as a COMT inhibitor Supplementary Table S4 of [39].

HRH1 has been identified as an alternative receptor for SARS-CoV-2 since it is directly bound to the N-terminal domain of viral spike proteins. When mice infected with SARS-CoV-2 were treated with antihistamines, pathological lung inflammation was mitigated. That study suggested the potential inhibitory effect of six first-generation antihistamines and five second-generation antihistamines [40]. Altogether, those evidence suggest the need to repurpose the role of antihistamines in the treatment of SARS-CoV-2 [41].

### 4.3. Limitations of the Study

#### 4.3.1. Comorbidity

We previously had detailed that up to 91 different drugs were identified in the first patients belonging to our institution and admitted to our reference hospital during the first wave [16]. Among those suffering just one pathology, 16.7% were treated with analgesics, benzodiazepine treatments or proton inhibitors; 13.3% received diuretics or ACEI, 6.7% received serotonin reuptake inhibitors, calcium, betablockers, other antiarrhythmics, direct thrombin inhibitors, or ophthalmic prostaglandin analogs; and 3% received another 13 different chronic drugs. Altogether, the variability in the pathologies, the reduced number of patients per group if they were considered separately, and the clear relationship of survival with polypharmacy, that was also correlated with age and not to a concrete drug or pathology, led us to group the pandemic patients depending on the number of chronic treatments here.

The details about the relationship between polypharmacy, age and hospital admissions in our area have been also detailed previously for the different COVID-19 variants [17] and are consistent with previous studies [19,20].

#### 4.3.2. Cases, Test Availability and Diagnosis Protocols

This study has been limited by the lack of precise quantification of cases in the first wave, due to the unavailability of diagnostic tests for the early detection of cases in primary care until June 2020. The multiple points of vaccination in 2021 for people younger than 60 years old may affect the CST register of the vaccination rates. However, although the overall vaccination is probably under-recorded, vaccination rates at public primary care centers in early 2021 were over 90% in those older than 60 years, in whom COVID-19 mortality is high. This allows for a good comparison of the overall mortality rate.

Diagnostic tests were available for hospital admissions from early in the COVID-19 pandemic, and all patients admitted with respiratory symptoms were tested [42] and were also considered to have COVID-19 if they showed bilateral interstitial pneumonia (which is rare in other illnesses). In primary care, however, tests were only available after the first wave, from June 2020, and the World Health Organization recommended ending active searches of infection in suspected cases from 24 March 2022 [43]. This meant that only cases identified between 1 June 2020 and 23 March 2022, were included. Furthermore, antigen tests were available to the public via pharmacies from autumn 2020 and were used in primary care centers; however, their sensitivity was reported to be only 78% in the first week of symptoms [44]. The absolute number of infections must, therefore, be interpreted with care. It is likely that the low sensitivity probably affects both groups equally, although it is uncertain if patients with known pathologies requiring antihistamines tested themselves earlier to avoid illness progression, thereby introducing selection bias. For those reasons, the infection, hospital admission, and mortality rates were calculated separately in the present report.

#### 4.3.3. Socioeconomic Factors

Life expectancy is known to be related to socioeconomic factors (79 years at metropolitan ‘ABS Prat de Llobregat 3’ or ‘St Adrià del Besós 2’ vs. 87 years in the residential area of the ‘ABS St Cugat del Vallès 2’). However, the rural factor, correlated with the distance to an advanced hospital, is also a strong health determinant in Catalonia: St Quirze de Besora in the Pre-Pyrenees mountains, with 79.7 years of health expectancy, is at position 396/398 of life expectancy compared to the suburb ‘Turó de la Peira’, at the 2/398 position with a life expectancy of 86.7 years, in the Nou Barris district just beside the prestigious and public Hospital Vall d’Hebron in Barcelona city. The population served by the CST, the institution where this study was conducted, has a life expectancy that falls between the highest and lowest in Catalonia, ranging from 81.7 to 83.8 years across the different CST health areas [22].

Despite a variable number of people with low and high incomes in the different CST health areas, the rate of infection during the full diagnosis period was similar in all health areas (21% in Rubí3 compared to 27% in Rubí2). The existence of a second private hospital in the zone may affect the quantification of the hospitalized population, but probably equally affect the AntiHm and NOAntiHm groups, that show very high OR, in favor of the group with AntiHm treatment, in several comparisons of hospital admission and death.

Chronic prescriptions are probably not affected by the existence of other private health services because they are also recorded by the public health service due to cost discounts in chronic treatments. It is feasible to assume that any associated bias equally affects infection and hospital admission rates among patients either taking or not taking antihistamines. Finally, although chronic prescription does not necessarily imply daily consumption, patients with polypharmacy tend to use pill boxes with the full authorized prescription, increasing the likelihood that they take all authorized treatments daily.

### 4.4. Future Approaches and Possible COVID-19 Waves

Other chronic drugs, such amantadine, have also been suggested to be protective and are currently under study in the same observational trial NCT05504057. The observed effect of this drug in initial COVID infections [45], but also in randomized controlled trials involving the fatigue in post-COVID syndrome [46], is consistent with the early findings of the potential protective effects of another drug for the treatment of Parkinson’s disease [39]. However, fewer than 100 patients are currently receiving treatment with amantadine in our institution. A multicenter study with other collaborations is being sought to study the effect of this drug.

COVID-19 hospital admissions have tripled (from 16 patients per million to 62.2 per million) in Catalonia in June 2024 and SARS-CoV-2 is isolated in 16.8% of random samples of symptomatic primary care patients, due to the FLiRT subvariant [47], suggesting that the mutations of the virus are still able to produce sudden periodical increases in hospital admissions [17,48] and that the search of therapies is still of interest. The long-term effect of repeated infections is uncertain, since neurological impairment has been described [49], even after suffering a mild infection.

## 5. Conclusions

In conclusion, patients with chronic antihistamine prescriptions (alone or with polypharmacy) showed reduced infection, hospital admission, and mortality rates consistent with the results of previous descriptive studies of prospective experiences. This suggests the safety of chronic antihistamine treatment, its possible use as symptomatic treatment during the early stages of the COVID-19 infection, and the need to explore its effectiveness in a randomized controlled prospective trial.

## Figures and Tables

**Table 1:** Socioeconomic characteristics of the CST population and rate of infection. The basic healthcare area (ABS) in Terrassa and Rubí, belonging to the CST institution where this study was performed, are compared with the other six area in Catalonia with lower and higher life expectancy, together with the percentages of the population with lower and high incomes and incomplete primary education. The rate of infection in the assigned population is indicated in parenthesis beside the number of the assigned population. All areas showed vaccination rates of over 90% in the population of people over 60 years old treated with at least two chronic treatments.

Basic Healthcare Area (ABS)	Population 2024 (COV Infection)	Life Expectancy (2015)	Incomes <18,000 €	Incomes >100,000 €	Incomplete Primary Education	VAC in >60 + ≥2 nT
El Prat de Llobregat 3	12.996	79.0	66.8%	0.1%	32.6%	
St Adrià del Besós 2	17.579	79.2	71.5%	0.1%	34.5%	
St Quirze de Besora	4.834	79.7	72.6%	0.4%	20.1%	
CST ABS						
TERRASSA B	28,399 (24%)	81.7	69.6%	0.04%	26.6%	90.7%
RUBI 3	17,796 (21%)	82.0	63.1%	0.3%	22.3%	94.2%
TERRASSA F (+Matadapera)	37,164 (22 + 24%)	83.1	67.6%	1.4%	24.1%	93.3% (+92.5%)
RUBÍ 2 (+Castellbisbal)	41,100 (27 + 24%)	83.3	65.2%	0.3%	21.8%	92.7% (+93.3%)
TERRASSA A	22,972 (23%)	83.8	58.8%	1.1%	16.9%	92.3%
						
Sta Perpètua de Mogoda	25,821	86.7	62.2%	0.27%	25.1%	
Barcelona 8-C: Turó de la Peira	23,968	86.7	69.4%	0.2%	27.1%	
Sant Cugat del Vallès 2	33,911	87.0	45.0%	7.8%	5.8%	

**Table 2:** Average age +/− standard deviation in assigned population over and below 60 years old, related to the number of chronic treatments (nT), receiving or not receiving antihistamine treatment (NoAntiHm or AntiHm groups).

	No AntiHm				AntiHm			
	0–59		≥60		0–59		≥60	
MAN	Mean	+/−	Mean	+/−	Mean	+/−	Mean	+/−
NoVAC pre-infection	31.2	16.7	67.6	16.6	36.7	19.4	69.6	15.5
0 nT	28.5	16.3	64.7					
1 nT	40.5	13.6	64.6	14.9	30.5	14.3	63.5	14.4
2–7 nT	45.3	11.3	68.2	15.2	38.8	15.1	68.3	13.6
≥8 nT	50.0	8.5	74.4	14.1	51.1	14.2	73.4	14.4
VAC pre-infection	32.6	18.2	70.4	19.1	40.9	23.6	70.4	18.8
0 nT	25.9	16.6	66.5			19.0		
1 nT	42.0	14.6	67.2	17.2	33.5	18.0	66.5	17.3
2–7 nT	48.6	11.0	70.4	18.0	41.7	15.1	69.1	17.4
≥8 nT	52.4	7.0	74.1	11.5	51.4	11.1	72.4	11.9
WOMAN								
NoVAC	30.6	17.1	69.2	8.9	39.8	12.5	69.4	9.4
Pre-infection								
0 nT	26.8	16.9	66.2	7.2				
1 nT	37.8	13.0	65.7	6.3	33.4	13.5	62.0	2.9
2–7 nT	44.2	11.3	69.2	8.6	40.7	117	67.8	8.2
≥8 nT	51.2	7.2	76.0	9.3	49.3	7.5	72.2	10.2
VAC	33.6	17.3	72.0	8.4	41.2	13.7	71.1	7.8
pre-infection								
0 nT	26.7	16.1	67.2	7.2				
1 nT	38.8	14.2	67.9	7.0	32.0	14.3	68.6	8.2
2–7 nT	47.2	11.0	71.7	8.2	42.1	12.9	69.8	7.3
≥8 nT	52.2	7.2	75.9	8.1	51.2	7.8	72.8	8.0

**Table 3:** Vaccination records during the pandemic, including those vaccinated after the first infection, stratified by age below or ≥60 years old, gender, and number of chronic treatments.

	Males			Females		
	No Vaccine Record	Vaccinated	%	No Vaccine Record	Vaccinated	%
No AntiHm						
0–59						
0 nT	28,905	13,757	32.2%	21,372	14,321	40.1%
1 nT	2869	2185	43.2%	3667	3444	48.4%
2–7 nT	3217	3899	54.8%	3537	5267	59.8%
>8 nT	115	383	76.9%	106	431	80.3%
≥60						
0 nT	511	1159	69.4%	486	1136	70.0%
1 nT	185	994	84.3%	203	1069	84.0%
2–7 nT	545	6401	92.2%	747	8078	91.5%
>8 nT	127	2034	94.1%	159	2993	95.0%
AntiHm						
0–59						
1 nT	150	100	40.0%	154	161	51.1%
2–7 nT	332	385	53.7%	490	662	57.5%
>8 nT	12	39	76.5%	41	93	69.4%
≥60						
1 nT	4	6	60.0%	5	16	76.2%
2–7 nT	15	218	93.6%	28	374	93.0%
>8 nT	10	146	93.6%	21	310	93.7%
Other mortality (2585)						

**Table 4:** Polypharmacy, antihistamines and cases. Number of pandemic cases in relation to vaccination status prior to the first infection and number of chronic treatments (nT) in the population assigned to the CST on March 2020. Suspected non-hospitalized cases from before 1 June 2020 and reported cases after 23 March 2022 (‘later dx’) are detailed in a separate column. Significant OR are identified with a *.

	NOAntiHm			AntiHm			
	No Infection Record	Infection	Suspected or Later dx	No Infection Record	Infection	Suspected or Later dx	OR NOAntiHm/ AntiHm
NoVAC pre-infection							
0	36,857	15,389	2636				
1	4653	2402	660	228	95	26	1.14
2–4	4617	2449	885	480	259	72	0.96
5–7	956	723	293	114	82	29	1.01
≥8	271	437	225	50	62	29	1.07 *
VAC pre-infection							
0	19,738	5629	1398				
1	4965	1328	608	153	68	27	0.70 *
2–4	10,594	1934	1362	620	161	83	0.75 *
5–7	6117	841	920	448	86	70	0.75 *
≥8	4128	570	717	402	66	63	0.85 *
Other mortality (2585)							

**Table 5:** Hospital admissions, death and polypharmacy. COVID-19 hospital admissions and related deaths stratified by population with several levels of polypharmacy (nT  =  number of chronic treatments prescribed) and vaccination status prior to the first infection. Significant results <0.05 are indicated by a *. Significancy after a Benjamini–Hochberg correction for múltiple comparisons is indicated by ‘+’.

	NoVAC Pre-infection		VAC Pre-infection		No Antihm/Antihm	
	NoAntiHm	AntiHm	NoAntiHm	AntiHm	OR noVAC	OR Vac
0 nT	54,882		26,765			
Hospital admission	220		31			
Survival	216		30			
CoV death	4		1			
No admission	54,662		26,734			
1 nT	7715	349	6901	248		
Hospital admission	103	4	25	1	1.16	0.90
Survival	100	4	25	1		
CoV death	3	0	0	0		
No admission	7612	345	6876	247		
2–7 nT	9923	1036	21,768	1468		
Hospital admission	389	16	187	6	2.54	2.10
Survival	311	14	166	5	(*p* < 0.0008) *+	(*p* < 0.03) *
CoV death	78	2	21	1	4.07	1.42
No admission	9534	1020	21,581	1462	(*p* < 0.03) *+	
≥8 nT	933	141	5415	531		
Hospital admission	255	24	186	13	1.61	1.41
Survival	145	16	125	8	(*p* < 0.03) *+	(*p* < 0.11)
CoV death	110	8	61	5	2.08	1.20
No admission	678	117	5229	518	(*p* < 0.01) *+	
Other mortality (2585)						

## Data Availability

The original contributions presented in this study are included in the article/Supplementary Materials. Further inquiries can be directed to the corresponding author.

## Supplementary Materials

The following supporting information can be downloaded at: https://www.mdpi.com/article/10.3390/microorganisms12122589/s1.
