# Supplementary material for: COVID-19 in Relation to Chronic Antihistamine Prescription
Source: Microorganisms. 2024 Dec 13;12(12):2589. doi: 10.3390/microorganisms12122589 (PMC11676390; doi:10.3390/microorganisms12122589)
Supplement: Supplementary file 1 [file microorganisms-12-02589-s001.zip › microorganisms-3348768-supplementary.pdf]

**Table S1.** COVID-19 Hospital admissions, death in hospital admitted patients and polypharmacy (nT = number of chronic treatments prescribed) related to age <>60 years old and vaccination prior to the first infection (VAC or NoVAC). Significant results <0.05 are indicated by a *. Significancy after a Benjamini-Hochberg correction for multiple comparisons is indicated by '+'.When COV death was 0 in some groups, the OR quotient was indicated as !

| **AntiHm Treatment** | **No AntiHm** | | **Total No AntiHm** | **AntiHm** | | **Total AntiHm** | **OR NoAntiHm/ /AntiHm** | | |
|---|---|---|---|---|---|---|---|---|---|
| **Age** | **0-59** | **>=60** | | **0-59** | **>=60** | | **0-59** | **>60** | **total** |
| **NoVAC** | 68958 | 4527 | 73485 | 1367 | 159 | 1526 | | | |
| **0nT** | 53737 | 1145 | 54882 | | | | | | |
| Hospital admission | 189 | 31 | 220 | | | | | | |
| Survival | 189 | 27 | 216 | | | | | | |
| COV death | | 4 | 4 | | | | | | |
| No hospital admission | 53548 | 1114 | 54662 | | | | | | |
| **1nT** | 7204 | 511 | 7715 | 336 | 13 | 349 | | | |
| Hospital admission | 80 | 23 | 103 | 2 | 2 | 4 | 1.86 | 0.29* | 1.16* |
| Survival | 79 | 21 | 100 | 2 | 2 | 4 | | | |
| COV death | 1 | 2 | 3 | 0 | 0 | 0 | ! | ! | ! |
| No hospital admission | 7124 | 488 | 7612 | 334 | 11 | 345 | | | |
| **2-7nT** | 7718 | 2205 | 9923 | 958 | 78 | 1036 | | | |
| Hospital admission | 128 | 261 | 389 | 9 | 7 | 16 | 1.76* | 1.32* | 2.54*+ |
| Survival | 123 | 188 | 311 | 9 | 5 | 14 | | | |
| COV death | 5 | 73 | 78 | **0** | 2 | 2 | ! | 1.29 | 4.07*+ |
| No hospital admission | 7590 | 1944 | 9534 | 949 | 71 | 1020 | | | |
| **>=8nT** | 296 | 637 | 933 | 73 | 68 | 141 | | | |
| Hospital admission | 17 | 238 | 255 | 6 | 18 | 24 | 0.69 | 1.41* | 1.61*+ |
| Survival | 14 | 131 | 145 | 6 | 10 | 16 | | | |
| COV death | 3 | 107 | 110 | **0** | 8 | 8 | ! | 1.43 | 2.08*+ |
| No hospital admission | 279 | 399 | 678 | 67 | 50 | 117 | | | |
| Mortality | 3 | 29 | 32 | | | | | | |
| **VAC** | 38520 | 22329 | 60849 | 1253 | 994 | 2247 | | | |
| **0nT** | 24618 | 2147 | 26765 | | | | | | |
| Hospital admission | 22 | 9 | 31 | | | | | | |
| Survival | 22 | 8 | 30 | | | | | | |
| COV death | | 1 | 1 | | | | | | |
| No hospital admission | 24596 | 2138 | 26734 | | | | | | |
| **1nT** | 4961 | 1940 | 6901 | 230 | 18 | 248 | | | |
| Hospital admission | 7 | 18 | 25 | **0** | 1 | 1 | ! | 0.17* | 0.89 |
| Survival | 7 | 18 | 25 | **0** | 1 | 1 | | | |
| No hospital admission | 4954 | 1922 | 6876 | 230 | 17 | 247 | | | |
| **2-7nT** | 8202 | 13566 | 21768 | 911 | 557 | 1468 | | | |
| Hospital admission | 26 | 161 | 187 | 2 | 4 | 6 | 1.44 | 1.65 | 2.10* |
| Survival | 24 | 142 | 166 | 2 | 3 | 5 | | | |
| COV death | 2 | 19 | 21 | **0** | 1 | 1 | ! | 0.78 | 1.42 |
| No hospital admission | 8176 | 13405 | 21581 | 909 | 553 | 1462 | | | |
| **>=8nT** | 739 | 4676 | 5415 | 112 | 419 | 531 | | | |

| | | | | | | | | | |
|---|---|---|---|---|---|---|---|---|---|
| Hospital admission | 9 | 177 | 186 | **0** | 13 | 13 | ! | 1.22 | 1.41 |
| Survival | 7 | 118 | 125 | **0** | 8 | 8 | | | |
| COV death | 2 | 59 | 61 | **0** | 5 | 5 | ! | 1.06 | 1.20 |
| No hospital admission | 730 | 4499 | 5229 | 112 | 406 | 518 | | | |
